# Blasius–Rayleigh–Stokes Flow of Hybrid Nanomaterial Liquid Past a Stretching Surface with Generalized Fourier’s and Fick’s Law

**DOI:** 10.3390/nano12030439

**Published:** 2022-01-27

**Authors:** Yingzi Jiang, Juan Zhang, Thabet Abdeljawad, Shafiq Ahmad, Muhammad Naveed Khan, Aysha Rehman, Abdulrazak H. Almaliki, Ahmed S. El-Shafay

**Affiliations:** 1 School of Mathematics and Statistics, Xuzhou Institute of Technology, Xuzhou 221018, China; xzitjyz@163.com; 2 Guangdong ATV Vocational College for the Performing Arts, Dongguan 523710, China; zj801106@163.com; 3 Department of Mathematics and Sciences, Prince Sultan University, Riyadh 11586, Saudi Arabia; 4 Department of Medical Research, China Medical University, Taichung 40402, Taiwan; 5 Department of Mathematics, Quaid-I-Azam University, Islamabad 44000, Pakistan; mnkhan@math.qau.edu.pk; 6 Department of Mathematics, University of Gujrat, Gujrat 50700, Pakistan; aysharehman1986@gmail.com; 7 Department of Civil Engineering, College of Engineering, Taif University, P.O. Box 11099, Taif 21944, Saudi Arabia; a.almaliki@tu.edu.sa; 8 Department of Mechanical Engineering, College of Engineering, Prince Sattam Bin Abdulaziz University, Alkharj 16273, Saudi Arabia; a.abdou@psau.edu.sa; 9 Mechanical Power Engineering Department, Faculty of Engineering, Mansoura University, Mansoura 35516, Egypt

**Keywords:** Blasius–Rayleigh–Stokes flow, hybrid nanofluid, generalized Fourier’s and Fick’s law, transitive magnetic field

## Abstract

The effect of Stefan blowing on the Cattaneo–Christov characteristics of the Blasius–Rayleigh–Stokes flow of self-motive Ag-MgO/water hybrid nanofluids, with convective boundary conditions and a microorganism density, are examined in this study. Further, the impact of the transitive magnetic field, ablation/accretion, melting heat, and viscous dissipation effects are also discussed. By performing appropriate transformations, the mathematical models are turned into a couple of self-similarity equations. The bvp4c approach is used to solve the modified similarity equations numerically. The fluid flow, microorganism density, energy, and mass transfer features are investigated for dissimilar values of different variables including magnetic parameter, volume fraction parameter, Stefan blowing parameter, thermal and concentration Biot number, Eckert number, thermal and concentration relaxation parameter, bio-convection Lewis parameter, and Peclet number, to obtain a better understanding of the problem. The liquid velocity is improved for higher values of the volume fraction parameter and magnetic characteristic, due to the retardation effect. Further, a higher value of the Stefan blowing parameter improves the liquid momentum and velocity boundary layer thickness.

## 1. Introduction

At present, researchers are keen to study heat transfer applications involving nanofluids. Based on empirical results in various technical and medical fields, the heat transport, mass, and density during the flow have dynamic features. Many base liquids are not favorable for heat transfer applications due to poor thermal effectiveness. To overcome this problem and improve the heat transfer efficiency, we added nanoparticles to the base fluids. The nano-size particles are most effective in enhancing the heat transfer rate. These nanoparticles consist mostly of metals such as silver, steel, gold, copper, potassium, nitrides, and many others. Choi [1] was the first to introduce the theory of nanofluids. Later, Buongiorno [2] discussed the concept of natural convective heat transport in a nanofluid. Sheikholeslami and Chamkha [3] explored the Lorentz force effect on the nanofluid flow. Ahmad et al. [4] introduced the influence of carbon nanotube nanoparticles on the boundary layer flow with thermal radiation, double stratification, and heat generation. They found that the thermal stratification diminished the temperature distribution. The natural convection heat transfer and inclined magnetic field effects of a molybdenum disulfide (MoS2) and magnesium oxide (MgO) nanofluid were investigated by Hymavathi et al. [5] via a vertical stretched surface embedded in a porous medium with a non-uniform heat source or sink. Nadeem et al. [6] used a magnetic dipole with three different ferrite nanoparticles to assess the effects of heat transport phenomena in a ferrofluid. They discovered that the characteristic of magneto-thermomechanical cooperation reduced the movement of liquid molecules, and as a result, increased the coefficient of skin friction and the thermal transport rate at the surface. Together with the impacts of viscous dissipation and non-linear thermal radiative effects, Reddy et al. [7] investigated the role of gyrotactic microorganisms in the mass and heat transport for the time-dependent MHD flow of a cross liquid mediated through nanoparticles. Vajravelu et al. [8] evaluated the influence of the nanoparticle volume fraction on the flow and heat transfer characteristics of Ag–water and Cu–water nanofluids under the impact of internal heat absorption or generation and thermal buoyancy. Jamshed et al. [9] explored the Casson non-Newtonian Cu–water and TiO_2_–water nanofluid flows in terms of heat transport and entropy generation under the influence of solar thermal transfer and slip conditions. Many researchers (see [10,11,12]) have recently observed an improvement in the heat transport rate in the presence of various nanoparticles. 

Many investigators have studied nanofluids to date, but studies related to hybrid nanofluids are attracting the attention of numerous scientists due to a wider range of heat conductivity applications. The combination of two or more nanoparticles in the base liquid generates a hybrid nanomaterial liquid. The reason that researchers are giving attention to this issue is the heat transport augmentation that can be accomplished by these hybrid nanofluids at a low cost of production. Mingzheng et al. [13] demonstrated the viscosity and thermal conductivity of various kinds of surfactant mixtures. Ahmad et al. [14] explored the thermal transport characteristics of a hybrid nanomaterial liquid with activation energy on a wedge, with the availability of variable velocity, Darcy–Forchheimer flow, and thermal slip. The influence of suction and buoyancy force effects on a Ag–MgO/water hybrid nanofluid flow across a stretching surface was introduced by Anuar et al. [15]. Maskeen et al. [16] explored the thermal transport properties of magneto-hydrodynamic hybrid alumina–copper/water nanomaterial liquid flow on an extending cylinder, with the influence of radiation and Joule heating. Ghalambaz et al. [17] demonstrated the thermal transport enhancement in a hybrid (Ag + MgO) nanofluid past a porous square cavity, using the local thermal non-equilibrium (LTNE) model. Manna et al. [18] proposed a new multi-banded magnetic field approach for improving the controllability of convective transports. Applying four equal parts of a magnetic field horizontally over a heated system occupied by porous material saturated with Cu–Al_2_O_3_/water hybrid nano liquid demonstrates the multi-banding approach. Ahmad and Nadeem [19] examined the properties of mass and heat transport in the presence of hybrid SWCNT–MWCNT/water nanomaterial liquid with Hall slip, ion slip, and chemical reaction impacts. Esfe et al. [20] scrutinized the impact of the volume fraction of nanoparticles on the dynamic viscosity and thermal conductivity of Ag–MgO/water hybrid nanofluids with particle sizes of 25(Ag) and 40(MgO) nm and nanofluid volume fractions (50 percent Ag and 50 percent MgO by volume) ranging from 0 to 2 percent and found new interrelations. In a modified Buongiorno’s model for the nanomaterial liquid magneto-transport phenomenon over an expanding cylinder in the vicinity of motile microorganism density, Rana et al. [21] integrated the Cattaneo–Christov mass flux (non-Fick’s) and heat (non-Fourier’s) ideas. Ma et al. [22] provided a (2D) numerical simulation of Ag–MgO nanomaterial forced convection and thermal transport in a channel with active coolers and heaters, to investigate the effect of a magnetic field on heat transport and nanomaterial liquid Ag–MgO forced convection.

Mass and heat transport phenomena occur due to the temperature and mass gradient respectively. Heat and mass transport have many industrial and engineering applications such as in heat exchangers, nuclear plants, heat pumps, heat conduction in tissues, refrigeration, heat transfer through materials, the diffusion of chemical impurities in rivers and oceans, the evaporation of water, etc. The convectional laws for the analysis of mass and heat transport used were Fourier’s [23] and Fick’s [24] laws. Fourier’s and Fick’s laws have a disadvantage; they give a parabolic type of energy and mass equation. To overcome this drawback, Cattaneo [25] modified Fick’s and Fourier’s laws by the addition of a time factor. Later, Christov [26] altered the Cattaneo law by replacing the time factor with the Oldroyd-B upper convective derivative. Han et al. [27] investigated the Maxwell liquid flow on a stretchable surface to present an evaluation of the Fourier heat flux and the Cattaneo–Christov model. With the addition of chemical reactions, a uniform heat source/sink, and thermal radiation, Venkateswarlu et al. [28] explored the impacts of a magnetic field on the flow of the Cattaneo–Christov heat flux model for MoS2 and MgO water-based nanofluids across a stretching sheet. Ali et al. [29] introduced the impact of the Cattaneo–Christov characteristics and bioconvection on self-motivated microorganisms in water-based nanoparticles with a leading-edge accretion/ablation and the Stefan blowing effect. The heat transport with respect to the Cattaneo–Christov concept with variable thermal relaxation time past a stretching sheet was discussed by Ahmad et al. [30]. Raju et al. [31] illustrated the heat, flow, and transport of mass attributes of a Maxwell nanofluid through a cylinder with a heat sink/source, using the Cattaneo–Christov premise. The Cattaneo–Christov premise was utilized by Malik et al. [32] to investigate the performance of Sisko fluid via a porous non-linearly stretched cylinder. Using a simplified mathematical model published by Jamshed and Aziz [33], the entropy production and heat transfer analysis of a thermal system containing hybrid nanomaterial with a Cattaneo–Christov flow model and thermal radiation impacts was explored. Garia et al. [34] used an extending surface with Joule heating and thermal radiation to solve the magneto-hydrodynamic flow of a SiO_2_−MoS_2_/water hybrid nanofluid past a wedge and a cone, with a generalized Fourier’s model.

The transport of electric current through any conducting material produces Joule heating. The collision of moving particles is the reason behind this. As a result of this process, some kinetic energy is transformed to heat, and the temperature of the material rises. Scientists and engineers have been fascinated by the idea of improving the efficiency of numerous mechanical systems and industrial machinery in recent years. Such problems can be overcome by lowering the temperature induced by Ohmic dissipation or Joule heating. As a result, several academics studied the flow issues from diverse physical perspectives. Sahoo [35] explored the impacts of partial slip and Joule heating on the flow of second-grade MHD liquid on a stretched surface with thermal transmission. Shehzad et al. [36] reported the radiative MHD extended flow of a Jeffrey liquid with Joule heating. Waqas et al. [37] discussed the Carreau–Yasuda nanomaterial liquid flow past an extending/shrinking surface in the presence of Joule heating, motile microorganisms, and thermal radiation, under Robin’s conditions. In the presence of thermal radiation and Joule heating, Kumar et al. [38] evaluated the 3D Oldroyd-B nanofluid flow across a stretching sheet. Hayat et al. [39] investigated the 3D steady second-grade nanomaterial flow via a rotating disc, with Joule heating and heat generation/absorption. For the constant wall heat flux condition, Chakraborty et al. [40] critically evaluated the transport-of-heat performance related to thermally developed coupled-electron magneto-hydrodynamic flows towards narrow flow conduits, taking electrokinetic impacts into account. Chen [41] conducted a study to investigate the impacts of Joule heating and viscous dissipation on the thermal transport and momentum, for magneto-hydrodynamic (MHD) flow towards an extending surface in the presence of radiation and surface suction/blowing.

Technical engineers, designers, manufacturers, and developers are keen to study Blasius–Rayleigh–Stokes flow. To transform the time-dependent flow model’s partial differential equations into a similar form, a new type of transformation was developed by Na [42], which consists of the Blasius–Rayleigh–Stokes variables. Fang [43] analyzed the heat transportation in an unsteady flow with the implementation of Blasius–Rayleigh–Stokes variables on a flat surface. Todd [44] was the first to establish the concept of leading-edge accretion/ablation, which is based on the unstable boundary layer model. The influence of radiation, Joule heating, and the Cattaneo–Christov theory applied to a Blasius–Rayleigh–Stokes flow towards a transitive magnetic field was discussed by Reddy et al. [45]. Several scholars have published studies on the Blasius–Rayleigh–Stokes variables in recent decades [46,47].

The unsteady MHD stagnation point Blasius-Rayleigh-Stokes hybrid nanomaterial liquid flow with Cattaneo-Christov theory, Joule heating, and convective conditions effect over an extending surface were investigated. To the best of our knowledge, no study has yet investigated the Blasius–Rayleigh–Stokes flow of hybrid nanomaterial liquid with convective boundaries. The Blasius–Rayleigh–Stokes variables were applied to transport the flow model into a couple of ODEs. The bvp4c MATLAB approach [48,49] was applied to solve these multiple equations numerically. The evolving parameters are discussed graphically along with the velocity, microorganism density, temperature, and concentration distribution. Further, a tabulated analysis of physical quantities is presented. To validate the problem, a comparison table is presented.

## 2. Mathematical Modeling

**Figure 1:**
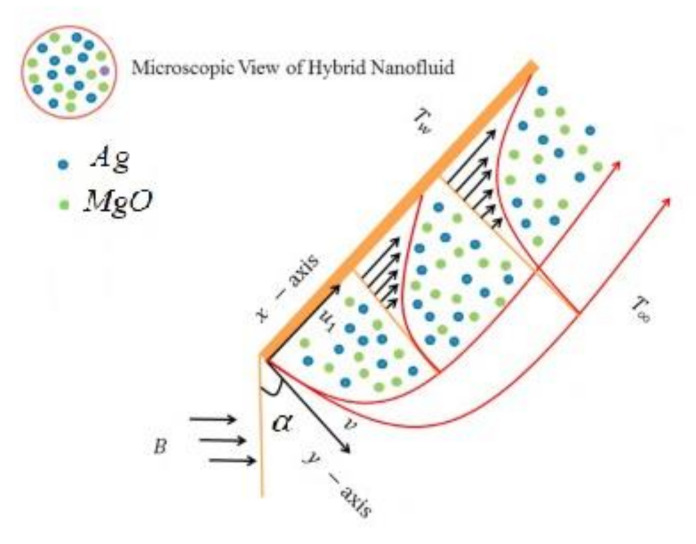
Problem configuration.

We consider the unsteady, laminar, incompressible, viscous, 2D, MHD boundary layer of the stagnation point Blasius–Rayleigh–Stokes flow of a hybrid nanofluid in the presence of viscous dissipation and microorganism density. The thermal and solutal energy transport analyses are presented with the influence of the Cattaneo–Christov theory and Joule heating. Furthermore, convective boundary conditions are applied on the surface of the stretching sheet and free-stream conditions are considered away from the surface. The transitive magnetic field is applied normally to the flow field. The free-stream and fluid velocities are $U_{\infty }$ and $U_{w}$, respectively, as shown in Figure 1. The fluid microorganism density, concentration, and temperature are N, C, and T, respectively. The wall temperature is $T_{w}$, the wall concentration is $C_{w}$, and the wall microorganism density is $N_{w}$; away from the wall these terms are $T_{\infty }$, $C_{\infty }$, and $N_{\infty }$, respectively. Using the boundary layer approximation of $O_{\left(u\right)\;}=1=O_{\left(x\right)},\;O_{\left(v\right)}=\delta =O_{\left(y\right)}$ and the above supposition, the equations of momentum, mass, energy, concentration, and microorganism density become [11,21,45]:

(1) $$\frac{\partial v}{\partial y}+\frac{\partial u}{\partial x}=0,$$

(2) $$\frac{\partial u}{\partial t}+v\frac{\partial u}{\partial y}+u\frac{\partial u}{\partial x}-U_{\infty }\frac{\partial U_{\infty }}{\partial x}-\frac{\partial U_{\infty }}{\partial t}=\upsilon_{hnf}\frac{\partial^{2}u}{\partial y^{2}}+\frac{\sigma_{hnf}B^{2}}{\rho_{hnf}}\sin^{2}\alpha u,\;$$

(3) $$\frac{\partial T}{\partial t}+\frac{\partial T}{\partial x}u+\frac{\partial T}{\partial y}v=\alpha_{hnf}^{*}\frac{\partial^{2}T}{\partial y^{2}}+\frac{\sigma_{hnf}B^{2}}{{\left(\rho C_{p}\right)}_{hnf}}\sin^{2}\alpha u^{2}+\frac{\mu_{hnf}}{{\left(\rho C_{p}\right)}_{hnf}}{\left(\frac{\partial u}{\partial y}\right)}^{2}\;-\lambda_{1}\left(\frac{\partial^{2}T}{\partial t^{2}}+\frac{\partial u}{\partial t}\frac{\partial T}{\partial x}+\frac{\partial v}{\partial t}\frac{\partial T}{\partial y}+u^{2}\frac{\partial^{2}T}{\partial x^{2}}+\left(u\frac{\partial v}{\partial x}+v\frac{\partial v}{\partial y}\right)\frac{\partial T}{\partial y}+v^{2}\frac{\partial^{2}T}{\partial y^{2}}+\left(v\frac{\partial u}{\partial y}+u\frac{\partial u}{\partial x}\right)\frac{\partial T}{\partial x}\right)\;-2\lambda_{1}\left(u\frac{\partial^{2}T}{\partial t\partial x}+v\frac{\partial^{2}T}{\partial t\partial y}+uv\frac{\partial^{2}T}{\partial x\partial y}\right),$$

(4) $$\begin{array}{l} \;\frac{\partial C}{\partial t}+\frac{\partial C}{\partial x}u+\frac{\partial C}{\partial y}v+\lambda_{2}\left(\frac{\partial^{2}C}{\partial t^{2}}+\frac{\partial u}{\partial t}\frac{\partial C}{\partial x}+\frac{\partial v}{\partial t}\frac{\partial C}{\partial y}+u^{2}\frac{\partial^{2}C}{\partial x^{2}}+v^{2}\frac{\partial^{2}C}{\partial y^{2}}\right)=D_{m}\frac{\partial^{2}C}{\partial y^{2}}\\ -2\lambda_{2}\left(u\frac{\partial^{2}C}{\partial t\partial x}+v\frac{\partial^{2}C}{\partial t\partial y}+uv\frac{\partial^{2}C}{\partial x\partial y}\right)-\lambda_{2}\left(u\frac{\partial v}{\partial x}+v\frac{\partial v}{\partial y}\right)\frac{\partial C}{\partial y}-\lambda_{2}\left(v\frac{\partial u}{\partial y}+u\frac{\partial u}{\partial x}\right)\frac{\partial C}{\partial x},\; \end{array}$$

(5) $$\;\frac{\partial N}{\partial t}+\frac{\partial N}{\partial x}u+\frac{\partial N}{\partial y}v=-\frac{bW_{c}}{C_{w}-C_{\infty }}\frac{\partial }{\partial y}\left(N\frac{\partial C}{\partial y}\right)+D_{n}\frac{\partial^{2}N}{\partial y^{2}},\;$$

and the related boundary conditions are [29]:

(6) $$\left.\begin{array}{l} u=0,-k_{hnf}\frac{\partial T}{\partial y}=h_{f}(T_{w}-T),-D_{m}\frac{\partial C}{\partial y}=h_{g}(C_{w}-C),\;\\ -D_{n}\frac{\partial N}{\partial y}=h_{n}(N_{w}-N),v=v_{w}=\frac{D_{m}}{1-C_{w}}\left(\frac{\partial C}{\partial y}\right),\;\mathrm{as}\;y=0, \end{array}\right\}$$

(7) $$u\to U_{\infty },T\to T_{\infty },N\to N_{\infty },C\to C_{\infty },\;\;\mathrm{as}\;y\to \infty .$$

In the above equations, $\sigma_{inf}$ is the electrical conductivity of the nanofluid, the density of the nanomaterials liquid is $\rho_{inf}$, the kinematic viscosity of the nanomaterials liquid is $\upsilon_{inf}$, the magnetic field is B, the thermal diffusion coefficient is $\alpha_{inf}^{*}$, the microorganism concentration and diffusivity coefficient are $D_{m}$ and $D_{n}$, respectively, $h_{f}$, $h_{g}$, and $h_{n}$ are the heat, mass, and microorganism transport coefficient, respectively, $\lambda_{1}$ and $\lambda_{2}$ are the thermal and concentration time relaxations, respectively, $W_{c}$ signifies the cell swimming speed, and b symbolizes the chemotaxis constant.

### 2.1. Similarity Analysis

Using the similarity transformation [29,44,45]:

(8) $$\zeta =y/\sqrt{\cos(w^{*})\upsilon_{f}t+\sin(w^{*})\left(\frac{\upsilon_{f}x}{U_{\infty }}\right)},\;\theta \left(\zeta \right)=\frac{T-T_{\infty }}{T_{w}-T_{\infty }},g\left(\zeta \right)=\frac{C-C_{\infty }}{C_{w}-C_{\infty }},$$

(9) $$h\left(\zeta \right)=\frac{N}{N_{w}},u=u_{w}f^{\prime }(\eta ),v=\frac{\left(\nu_{f}/2)\left(\zeta f^{\prime }(\zeta )-f(\zeta )\right)\sin(w^{*}\right)}{\sqrt{\cos(w^{*})\upsilon_{f}t+\sin(w^{*})\left(\frac{\upsilon_{f}x}{U_{\infty }}\right)}},$$

Applying Equation (7), the above nanoparticle phase equation transmutes to:

(10) $$\frac{\mu_{r}}{\rho_{r}}f^{\prime \prime \prime }+\frac{1}{2}\cos(w^{*})\zeta f^{\prime \prime }+\frac{1}{2}\sin(w^{*}){ff}^{\prime \prime }+\frac{M\sigma_{r}}{\rho_{r}}\sin^{2}\alpha (1-f^{\prime })=0,$$

(11) $$\begin{array}{c} \frac{k_{r}}{{\left(\rho C\right)}_{r}}\theta^{\prime \prime }+\frac{\mathrm{Pr}}{2}\left(\zeta \theta^{\prime }\cos(w^{*})+f\sin(w^{*})\theta^{\prime }+\gamma_{e}\sin(w^{*})\left(3{ff}^{\prime }\theta^{\prime }+\zeta f\theta^{\prime \prime }\right)\right)\\ +\frac{E_{c}M\sigma_{r}}{{\left(\rho C\right)}_{r}}\sin^{2}\alpha {f^{\prime }}^{2}+\frac{E_{c}\mu_{r}}{{\left(\rho C\right)}_{r}}{f^{\prime \prime }}^{2}=0, \end{array}$$

(12) $$g^{\prime \prime }+\frac{\mathrm{Pr}L_{e}}{2}\left(\zeta \cos(w^{*})g^{\prime }+f\sin(w^{*})g^{\prime }+\gamma_{c}\sin(w^{*})\left(3{ff}^{\prime }g^{\prime }+\zeta {fg}^{\prime \prime }\right)\right)=0,$$

(13) $$h^{\prime \prime }+\frac{\mathrm{Pr}L_{b}}{2}\left(\zeta \cos(w^{*})h^{\prime }+f\sin(w^{*})h^{\prime }\right)-P_{e}\left({hg}^{\prime \prime }+h^{\prime }g^{\prime }\right)=0,$$

The transformed conditions are:

(14) $$\left.\begin{array}{l} f^{\prime }(0)=0,f(0)=\frac{2s}{L_{e}\mathrm{Pr}\sin w^{*}}g,\;k_{r}\theta^{\prime }(0)=-B_{e}(1-\theta (0)),\\ g^{\prime }(0)=-B_{c}(1-g(0)),h^{\prime }(0)=-B_{n}(1-h(0)), \end{array}\right\}$$

(15) $$f^{\prime }(\zeta )\to 1,\theta (\zeta )\to 0,\;g(\zeta )\to 0,h(\zeta )\to 0,\;\mathrm{at}\;\zeta \to \infty ,$$

The parameters ablation/accretion, thermal Biot number, Lewis number, concentration Biot number, microorganism Biot number, magnetic field parameter, bio-convection Lewis number, Stefan blowing parameter, Eckert number, thermal relaxation parameter, concentration relaxation parameter, Peclet number, and solid volume fraction of particles are symbolized by $w^{*}$, $B_{e},\;L_{e},\;B_{c},\;B_{n},M,\;L_{b},\;s,\;E_{c},\;\gamma_{e},\gamma_{c}$, $P_{w}$ and $\phi_{1}$, respectively. These parameters are defined as:

(16) $$\gamma_{e}=\lambda_{1}U_{\infty },\gamma_{c}=U_{\infty }\lambda_{2},\mathrm{Pr}=\frac{\upsilon_{f}}{\alpha_{f}},L_{e}=\frac{\upsilon_{f}}{D_{m}},M=\frac{\sigma_{f}B_{0}{}^{2}}{\rho_{f}},$$

(17) $$L_{b}=\frac{\upsilon_{f}}{D_{n}},E_{c}=\frac{u_{w}{}^{2}}{C_{p}(T_{w}-T_{\infty })},\;s=\frac{\left(C_{w}-C_{\infty }\right)}{\left(1-C_{w}\right)},B_{t}=-\frac{h_{f}}{k_{f}}\sqrt{\frac{\upsilon_{f}}{\alpha_{f}}},$$

The hypothetical relation is characterized as follows [20,21,22].

(18) $$\sigma_{r}=\frac{\sigma_{hnf}}{\sigma_{f}}=\left[\frac{1+3\left(\frac{\sigma }{\sigma_{f}}-1\right)\left(\phi_{MgO}+\phi_{Ag}\right)}{\left(\frac{\sigma }{\sigma_{f}}+2\right)-\left(\frac{\sigma }{\sigma_{f}}-1\right)\left(\phi_{MgO}+\phi_{Ag}\right)}\right],$$

(19) $${\left(\rho c\right)}_{r}=\frac{{\left(\rho c\right)}_{hnf}}{{\left(\rho c\right)}_{f}}=\left(1+\phi_{MgO}-\phi_{Ag}\right)+\phi_{Ag}\frac{{\left(\rho c\right)}_{Ag}}{{\left(\rho c\right)}_{f}}+\phi_{MgO}\frac{{\left(\rho c\right)}_{MgO}}{{\left(\rho c\right)}_{f}},$$

(20) $$\rho_{r}=\frac{\rho_{hnf}}{\rho_{f}}=\left(1+\phi_{MgO}-\phi_{Ag}\right)+\phi_{Ag}\frac{\rho_{Ag}}{\rho_{f}}+\phi_{MgO}\frac{\rho_{MgO}}{\rho_{f}},$$

(21) $$\begin{array}{c} \mu_{r}=\frac{\mu_{hnf}}{\mu_{f}}=\left[1+32.795\phi_{1}-7214\phi_{1}^{2}+714600\phi_{1}^{3}-0.1941\times 10^{8}\phi_{1}^{4}\right];\;\\ \;\;\;\;\;\;\;\;\;\;\;0\leq \phi_{1}\leq 0.02, \end{array}$$

(22) $$\begin{array}{c} k_{r}=\frac{k_{hnf}}{k_{f}}=\left[\frac{0.1747\times 10^{5}+\phi_{1}}{\begin{array}{c} 0.1747\times 10^{5}-0.1498\times 10^{6}\phi_{1}+0.1117\times 10^{7}\phi_{1}^{2}\\ +0.1997\times 10^{7}\phi_{1}^{3} \end{array}}\right];\\ \;\;\;\;\;\;\;\;\;\;\;\;\;\;0\leq \phi_{1}\leq 0.03. \end{array}$$

### 2.2. Quantities of Interest

The local friction drag and the local density number of motile microorganisms are:

(23) $$C_{f}=\frac{\mu_{hnf}}{\rho_{f}u_{\infty }{}^{2}}{\left[\frac{\partial u}{\partial y}\right]}_{y=0},Nn_{x}=\frac{-x}{N_{w}}{\left[\frac{\partial N}{\partial y}\right]}_{y=0},$$

Using Equation (10), the dimensionless form is,

(24) $$\begin{array}{c} Re_{x}^{1/2}C_{fx}=\frac{\mu_{r}f^{\prime \prime }(0)}{\sqrt{\cos(w^{*})\tau +\sin(w^{*})}},Re_{x}^{-1/2}Nn_{x}=\frac{-h^{\prime }(0)}{\sqrt{\cos(w^{*})\tau +\sin(w^{*})}}. \end{array}$$

The local Reynolds number is characterized as $Re_{x}=\frac{xu_{\infty }}{v_{f}}$.

## 3. Numerical Method and Evidence

The MATLAB BVP4C functions are now used to obtain numerical solutions for non-linear coupled ODEs (10)–(13) with boundaries (14) and (15). The MATLAB BVP4C functions can only solve first-order ODEs. We transformed the second- and third-order DEs to first-order DEs and chose a reasonable value of $\eta_{\infty }$, where 10^−6^ was left aside as the absolute convergence threshold, and consequently the first-order classifications were:

(25) $$f^{\prime \prime }=y(3),f^{\prime }=y(2),f=y(1),\;\theta^{\prime }=y(5),\theta =y(4),g^{\prime }=(7),g=y(6),h^{\prime }=y(9),h=y(8),$$

(26) $$yy1=f^{\prime \prime \prime }=\frac{\rho_{r}}{\mu_{r}}\left\{-\frac{1}{2}\cos(w^{*})\zeta y(3)-\frac{1}{2}\sin(w^{*})y(1)y(3)-\frac{M\sigma_{r}}{\rho_{r}}\sin^{2}\alpha (1-y(2))\right\},$$

(27) $$yy2=\theta^{\prime \prime }=\frac{1}{\left\{\frac{k_{r}}{{\left(\rho C\right)}_{r}}+\frac{\mathrm{Pr}\gamma_{e}\zeta \sin(w^{*})y(1)}{2}\right\}}\left\{\begin{array}{l} -\frac{\mathrm{Pr}}{2}\left(\zeta \cos(w^{*})y(5)+\gamma_{e}\sin(w^{*})\left(3y(1)y(2)y(5)\right)\right)\\ -\frac{\mathrm{Pr}}{2}y(1)\sin(w^{*})y(5)-\frac{E_{c}M\sigma_{r}}{{\left(\rho C\right)}_{r}}\sin^{2}\alpha y{\left(2\right)}^{2}-\frac{E_{c}\mu_{r}}{{\left(\rho C\right)}_{r}}y{\left(3\right)}^{2} \end{array}\right\},$$

(28) $$yy3=g^{\prime \prime }=\frac{1}{\left(1+\frac{\mathrm{Pr}L_{e}\gamma_{c}\sin(w^{*})\zeta y(1)}{2}\right)}\left\{\begin{array}{l} -\frac{\mathrm{Pr}L_{e}}{2}\left(\zeta \cos(w^{*})y(7)+y(1)\sin(w^{*})y(7)\right)\\ -\frac{\mathrm{Pr}L_{e}}{2}\gamma_{c}\sin(w^{*})\left(3y(1)y(2)y(7)\right) \end{array}\right\},$$

(29) $$yy4=h^{\prime \prime }=-\frac{\mathrm{Pr}L_{b}}{2}\left(\zeta \cos(w^{*})y(9)+y(1)\sin(w^{*})y(9)\right)+P_{e}\left(y(8)yy3+y(9)y(7)\right),$$

with the conditions,

(30) $$\left.y_{0}(2);y_{0}(2)-\frac{2s}{L_{e}\mathrm{Pr}\sin w^{*}}y_{0}(6);\;k_{r}y_{0}(5)+B_{e}(1-y_{0}(4));y_{0}(7)+B_{c}(1-y_{0}(6));y_{0}(9)+B_{n}(1-y_{0}(8))\right\},$$

(31) $$y_{\inf}(2)-1;\;\;y_{\inf}(4);\;\;y_{\inf}(6);y_{\inf}(8);$$

In conclusion, $\eta_{\infty }=6$ is used in this study to attain the asymptotic values provided by the boundary condition (14) and (15). Because the relationship determines a high level of comprehension for each considered value, we are confident that this conclusion is correct and precise. Table 1 shows that Nanoparticles (Ag, MgO) and base fluid (water) thermo-physical characteristics. Table 2 is a comparative analysis for given values of $\omega^{*}$ (ablation/accretion parameter) against the velocity gradient. It can be seen that larger values of $\omega^{*}$ improve the velocity gradient.

## 4. Results and Discussion

**Figure 2:**
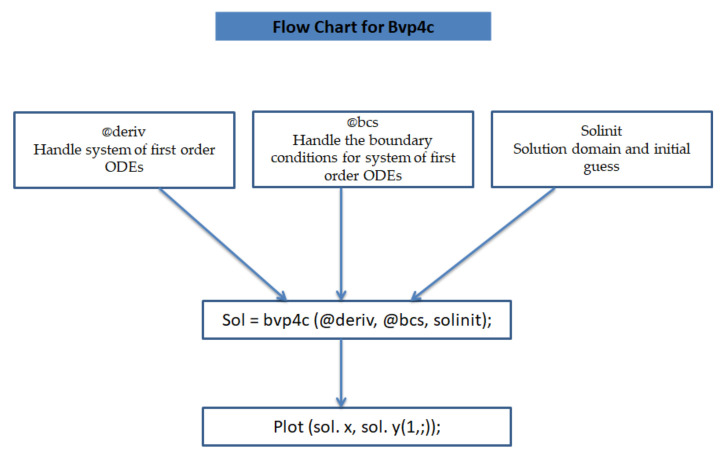
Flowchart of bvp4c scheme.

**Figure 3:**
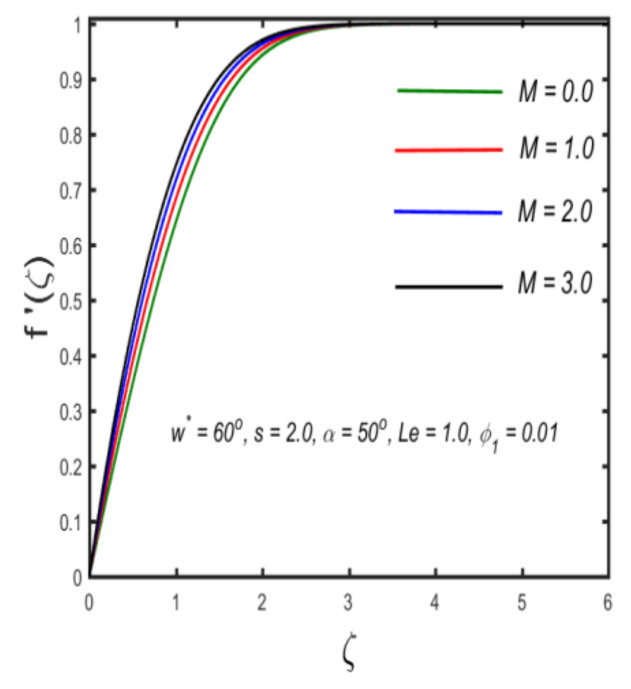
Variation in the velocity against magnetic parameter.

**Figure 4:**
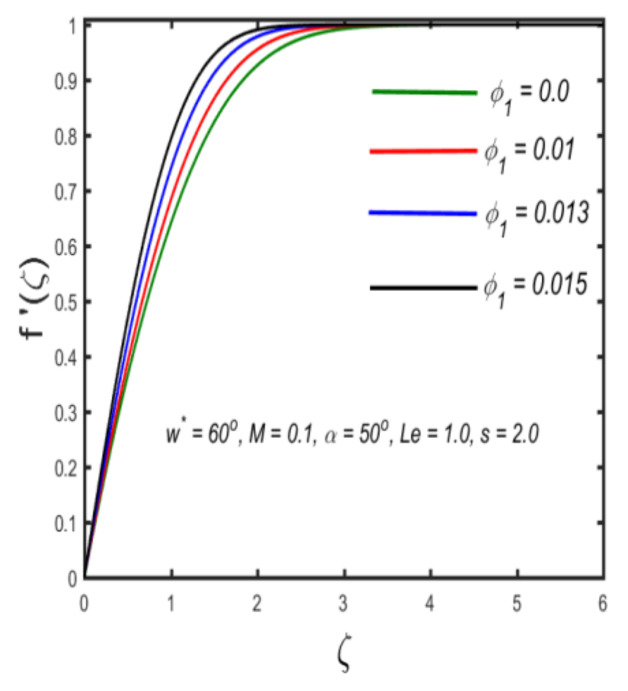
Variation in the velocity profile against solid volume fraction.

**Figure 5:**
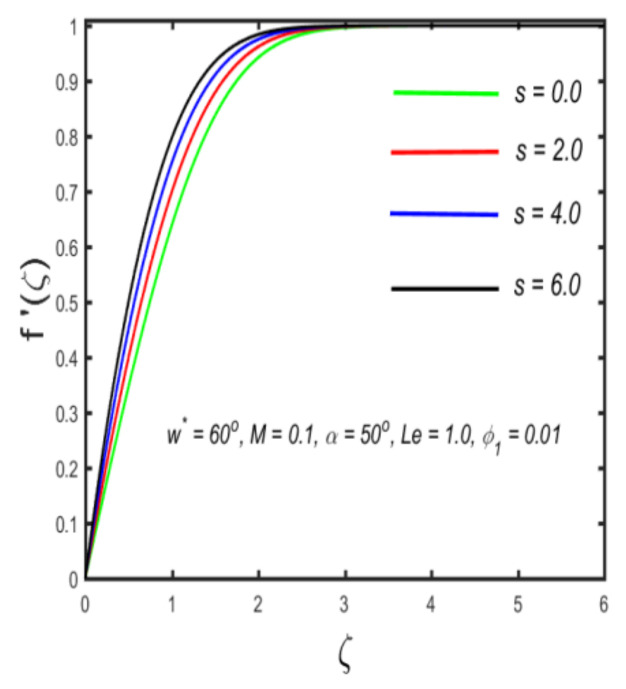
The effect of velocity along with Stefan blowing parameter.

**Figure 6:**
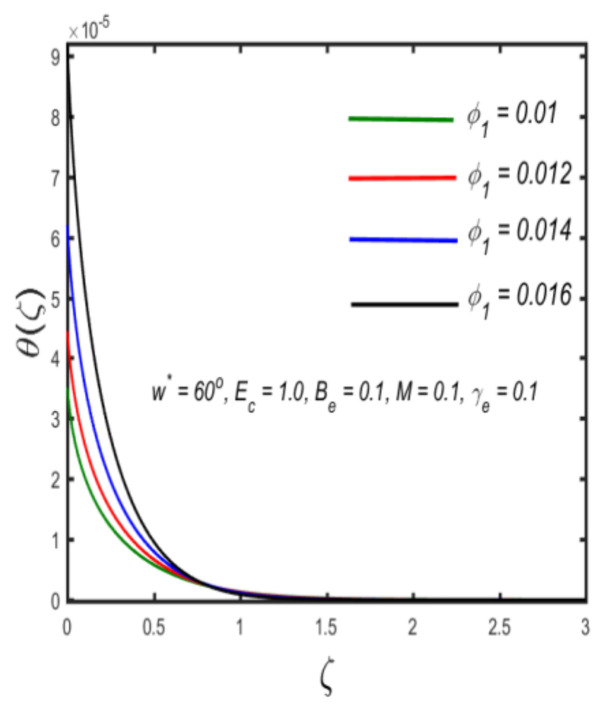
The effect of temperature against solid volume fraction.

**Figure 7:**
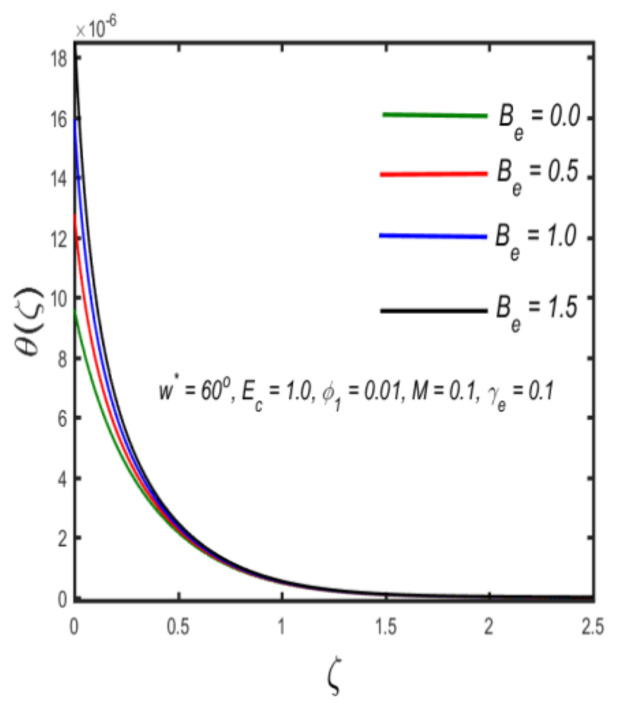
The effect of temperature against thermal Biot number.

**Figure 8:**
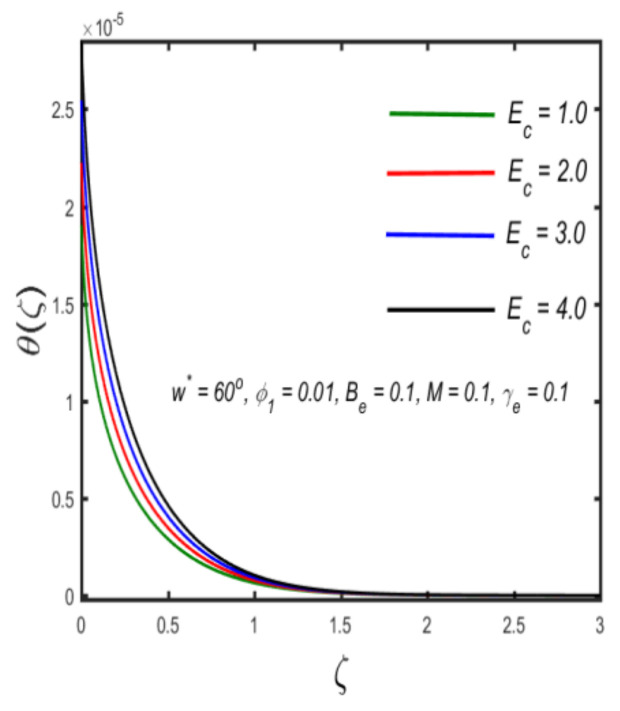
Variation in the temperature against Eckert number.

**Figure 9:**
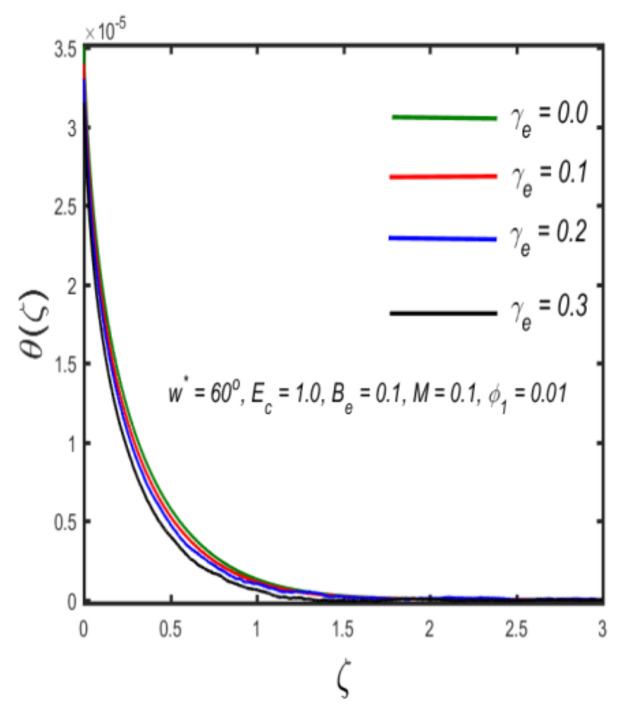
Variation in temperature against thermal relaxation parameter.

**Figure 10:**
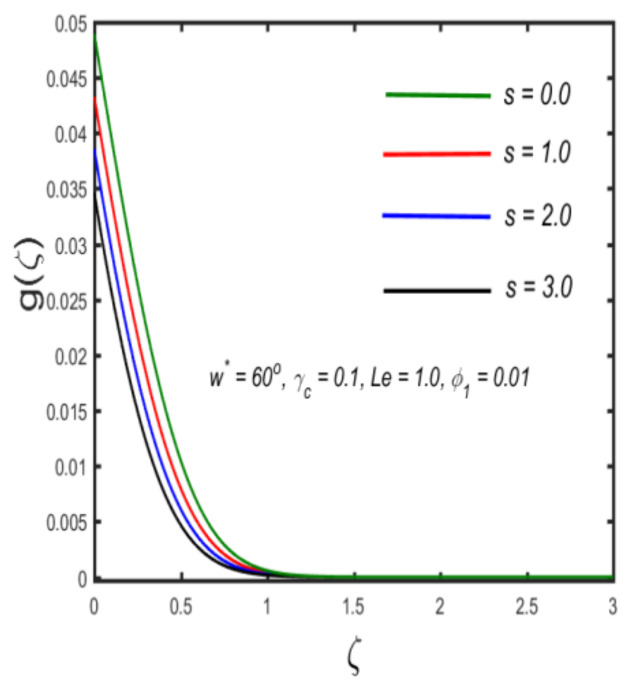
The effect of concentration against Stefan blowing parameter.

**Figure 11:**
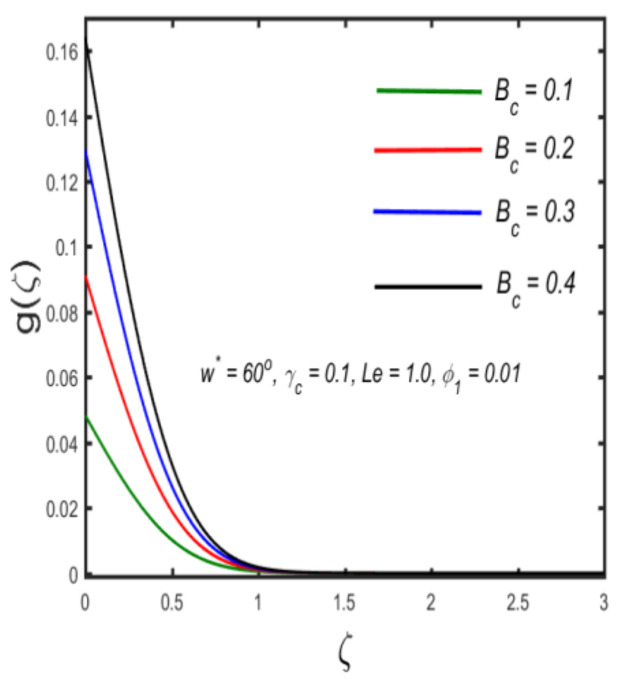
Graph of concentration against concentration Biot number.

**Figure 12:**
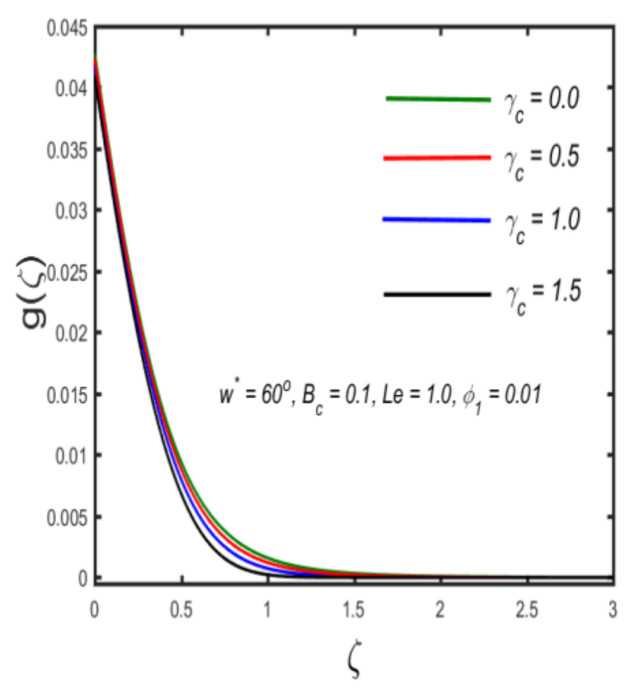
The effect of concentration against concentration relaxation parameter.

**Figure 13:**
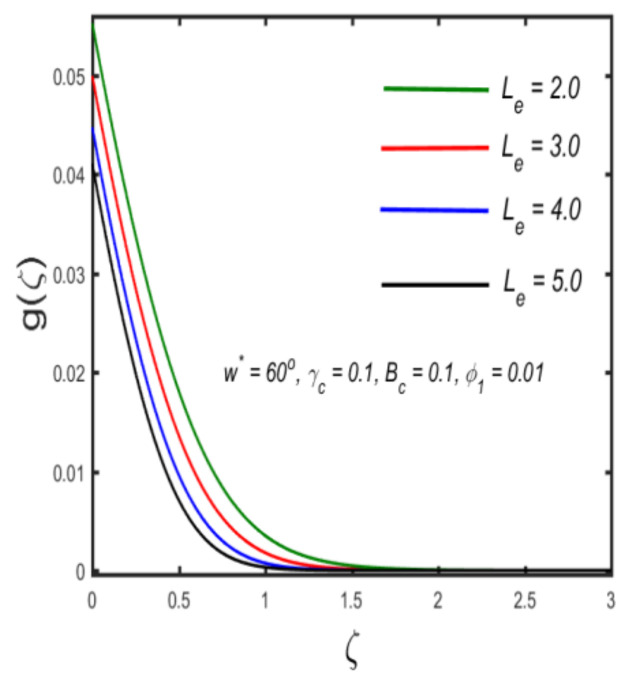
The effect of concentration against Lewis number.

**Figure 14:**
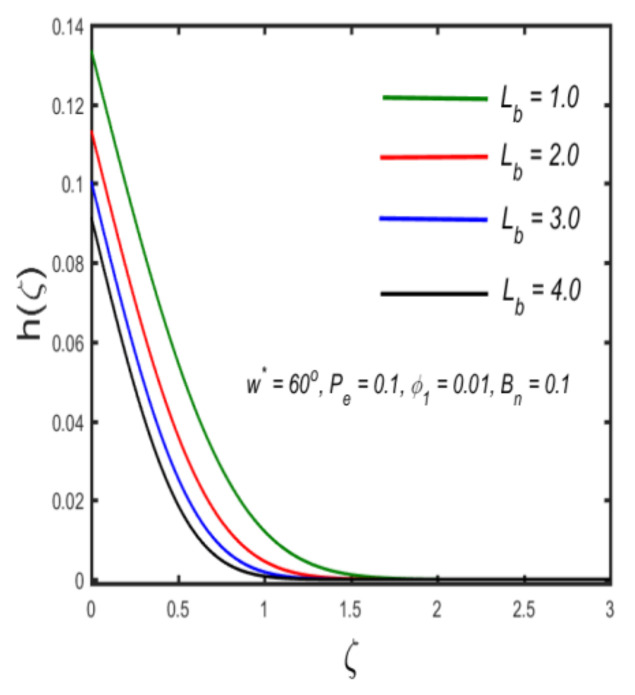
The effect of the density of motile microorganisms against bio-convection Lewis parameter.

**Figure 15:**
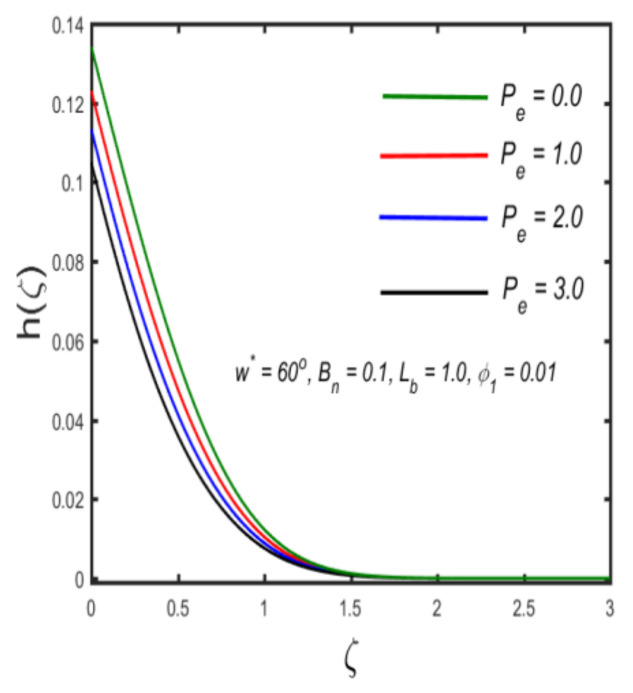
The effect of the density of motile microorganisms against Peclet number.

**Figure 16:**
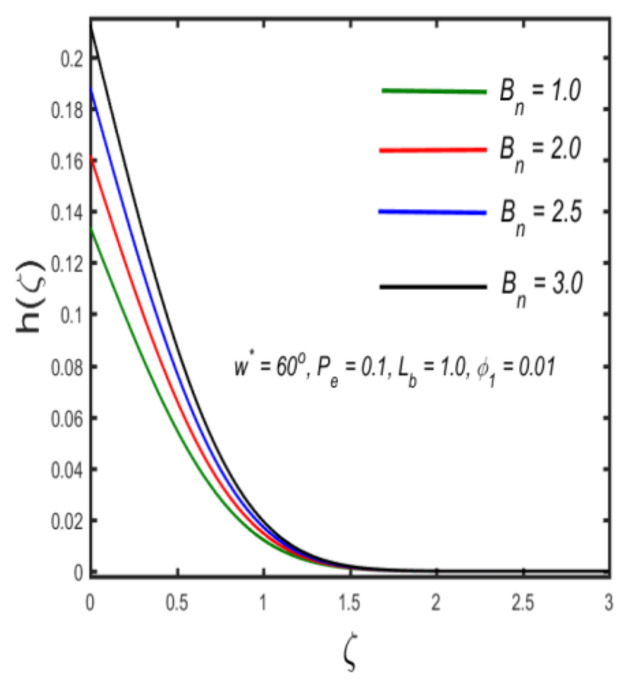
The effect of the density of motile microorganisms against microorganism Biot number.

In this section, the physical implications of prominent characteristics such as the magnetic characteristic $\left(0\;\leq M\;\leq 3\right)$, Stefan blowing parameter $\left(0\;\leq s\;\leq 6\right)$, solid volume fraction $\left(0\;\leq \;\phi_{1}\;\leq 0.03\right)$, thermal Biot number $\left(0\;\leq \;B_{e}\;\leq 1.5\right)$, Eckert number $\left(I\;\leq \;E_{c}\;\leq 4\right)$, thermal relaxation parameter $\left(0\;\leq \;\gamma_{e}\;\leq 0.3\right)$, concentration Biot number $\left(0.1\;\leq \;B_{c}\;\leq 0.4\right)$, Lewis number $\left(1\;\leq \;L_{e}\;\leq 5\right)$, concentration relaxation parameter $\left(0\;\leq \;\gamma_{c}\;\leq 1.5\right)$, bio-convection Lewis parameter $(1\;\leq \;L_{b}\;\leq 4$, Peclet number $\left(0\;\leq \;P_{e}\;\leq 3\right)$, and microorganism Biot number $\left(1\;\leq \;B_{n}\;\leq 3\right)$ against velocity, temperature, the volumetric concentration of nanoparticles, and motile microorganism number is reflected in Figures 3–16. Table 3 shows that higher values of $\phi_{1}$ increase the skin friction, whereas higher estimates of M, α and s reduce the skin-friction coefficient. The effect of distinct values of $L_{b},\;P_{e},\;B_{n}$ and $\omega^{*}$ along with the microorganism number is presented in Table 4. It was discovered that higher estimates of $L_{b},\;P_{e}$ and $\omega^{*}$ increase the motile density transfer rate; however, higher values of $B_{n}$ have the reverse effect. Figure 2 describes the flowchart of bvp4c scheme. The influence of the magnetic parameter (M)**,** volume fraction parameter ($\phi_{1}$), and Stefan blowing parameter (s) on the liquid velocity is indicated in Figure 3, Figure 4 and Figure 5. It can be noted from Figure 3 that a stronger estimate of M increases the velocity profile. The cause of this pattern is that a magnetic field in an electrically conducting liquid induces a Lorentz force and reduces the momentum boundary layer flow considerably. Figure 4 displays the influence of distinct values of the volume particle parameters versus velocity distribution, and we can perceive that the impact of solid volume fraction enhances thermal transmittance in both solutions, due to the increased number of nanoparticles in the fluid system, which increases resistance while also improving boundary layer thicknesses. Figure 5 displays the effect of the Stefan blowing characteristic on the fluid velocity. It can be noted that the fluid velocity is increased due to a higher estimate of s. Physically, buoyancy forces emerge as a result of the blowing effect, which intensify fluid movement and transport heated nanofluid away from the surface. The influence of the volume fraction parameter ($\phi_{1}$), thermal Biot number ($B_{e}$), Eckert number ($E_{c}$), and thermal relaxation characteristic ($\gamma_{e}$) on the thermal field is presented in Figure 6, Figure 7, Figure 8 and Figure 9. It can be seen from Figure 6 that larger values of $\phi_{1}$ increase the fluid temperature because, due to a larger $\phi_{1}$, a retardation effect takes place, which boosts the temperature. The effect of the Biot number on the temperature is described in Figure 7. The Biot number, in reality, includes the heat transfer coefficient. As the Biot number increases, so does the heat transmission coefficient. The fluid temperature and the related boundary layer thickness increase as the heat transport coefficient increases. Figure 8 depicts the features of $E_{c}$ in the temperature distribution. High Eckert numbers cause the fluid temperature to rise faster because the fluid particles move faster, allowing them to collide more frequently and produce heat, causing the fluid temperature to rise faster. Figure 9 shows that an increase in the value of the thermal relaxation characteristic reduces the temperature. Physically, this is because the time it takes for material particles to transfer heat to adjacent particles reduces when $\gamma_{e}$ is increased. In other words, greater values of $\gamma_{e}$ cause the material to behave in a non-conducting manner, resulting in a drop in fluid temperature. Furthermore, it should be highlighted that for $\gamma_{e}=\;0$, heat is quickly transferred throughout the material. The temperature profile is greater for $\gamma_{e}=\;0$, i.e., in the Fourier’s law case, than for the Cattaneo–Christov premise. The influence of the Stefan blowing parameter (s), concentration Biot number ($B_{c}$), Lewis number ($L_{e}$), and relaxation concentration characteristic ($\gamma_{c}$) on the concentration is described in Figure 10, Figure 11, Figure 12 and Figure 13. Figure 10 shows that a larger estimate of s reduces the concentration. The effect of $B_{c}$ on the concentration is shown in Figure 11. Fluid particles require a longer time to diffuse across the material medium as $B_{c}$ is increased. As a result, the concentration distribution is reduced. Figure 12 shows the varying behavior of $\gamma_{c}$ along the concentration distribution. It is drawn showing that a larger assessment of $\gamma_{c}$ reduces the concentration. Physically, more time is required to transport the nanoparticles from one place to another. The impact of the Lewis number on the concentration is seen in Figure 13. It is interesting to note that $L_{e}$ is the ratio between thermal and mass diffusivity; as we increase $L_{e}$, the mass diffusivity increases, and as a consequence the concentration profile declines. The influence of the bio-convection Lewis parameter ($L_{b}$), Peclet number ($P_{e}$), and microorganism Biot number ($B_{n}$) on the microorganism density is shown in Figure 14, Figure 15 and Figure 16. It can be seen from Figure 14 and Figure 15 that larger values of $L_{b}$ and $P_{e}$ reduce the microorganism density profile. Physically, with an increment in $L_{b}$ and $P_{e}$, the microorganism diffusivity declines, and as a result both curves are reduced. Figure 16 shows the behavior of $B_{n}$ against the microorganism density profile. It shows that the microorganism density profile is enhanced due toa larger estimation of $B_{n}$.

## 5. Concluding Remarks

The time-dependent 2D Blasius–Rayleigh–Stokes flow of hybrid nanomaterial liquid with magnetic field and microorganism effects is discussed. Convective conditions are applied at the surface of the extending surface. The main results of the current research are as follows:

1. The velocity of the liquid is increased for higher estimates of the volume fraction parameter and magnetic parameter, due to the retardation effect.
2. Higher values of the Stefan blowing parameter improve the velocity of the liquid and the momentum boundary layer thickness.
3. A decaying trend occurs due to a higher thermal relaxation characteristic because particles have extra time to transport heat to nearby particles.
4. Kinetic energy is transformed into heat energy due to the enhancement of the Eckert number.
5. The nanoparticle concentration declines due to larger estimates of concentration relaxation and Stefan blowing parameter.
6. A higher estimate of thermal and concentration Biot number improves the heat and mass transfer rates, respectively.
7. A decreasing behavior occurs in the microorganism density profile due to larger values of $L_{b}$ and $P_{e}$.
8. The motile density transfer rate decays for larger values of $B_{n}$.

## Figures and Tables

**Table 1:** Nanoparticles (Ag, MgO) and base fluid (water) thermo-physical characteristics [20,21,22].

Physical Properties	Base Fluid	Nanoparticle	
	Water	Ag	MgO
CpJkgK	4179.0	235	955
ρkgm3	997.10	10,500	3560
kWmK	0.620	429	45

**Table 2:** Comparative study of $f^{\prime \prime }\left(0\right)$ (velocity gradient) along with specific values of $\omega^{*}$ (ablation/accretion characteristic) when *s* = 0.

$\omega^{*}$	Mabood et al. [47]	Todd et al. [44]	Ali et al. [29]	Our Results
0	0.564189	0.5642	0.564190	0.564191
π24	0.575016	0.5750	0.575019	0.575020
π12	0.580728	0.5807	0.580726	0.580727
π6	0.577001	0.5770	0.577002	0.577003
π4	0.552875	0.5529	0.552876	0.552877
π3	0.507218	0.5072	0.507221	0.507222
5π12	0.436864	0.4369	0.436867	0.436868
11π24	0.389999	0.3900	0.390002	0.390003
π2	0.332057	0.3321	0.332057	0.332058

**Table 3:** The $f^{\prime \prime }\left(0\right)$ (local skin friction coefficient), along with other parameters.

$\phi_{1}$	M	α	s	Rex12Cf
0.0	1.0	50°	1.0	0.62880
0.01				0.68261
0.02				0.70860
	2.0			0.65672
	3.0			0.64121
	4.0			0.62612
		0°		0.26505
		30°		0.24703
		45°		0.22704
			0.2	0.34735
			0.3	0.32034
			0.4	0.30347

**Table 4:** $Re_{x}^{-1/2}\;Nn_{x}$, along with other parameters.

$L_{b}\;$	$P_{e}$	$B_{n}$	$\omega^{*}$	Rex12 Nnx
0.5	0.4	0.2	π4	3.84684
0.6				3.92359
0.7				3.99025
	0.1			3.49515
	0.3			3.58288
	0.5			3.67057
		0.4		3.83391
		0.5		3.81670
		0.6		3.80203
			5π12	3.88790
			11π24	3.92790
			π2	3.96792

## Data Availability

All data supporting this study are available in the article.

## Abbreviations

u, v	Velocity components	$B_{e}$	Thermal Biot number
x, y	Coordinates	$B_{c}$	Concentration Biot number
M	Magnetic parameter	$B_{n}$	Microorganism Biot number
B	magnetic field	$\omega^{*}$	Ablation/accretion parameter
$T,\;T_{w}$	Temperature, and wall temperature	Greek symbols	
$D_{n}$	Diffusivity of microorganisms	$\rho_{hnf},\;\rho_{f}$	Density
Pr	Prandtl number	$\mu_{hnf},\;\mu_{f}$	Dynamic viscosity
$C_{p}$	Specific heat	$\tau_{xy}$	Shear stress
$U_{w}$	Stretching velocity along the *x*-direction	$\alpha_{hnf},\;\alpha_{f}$	Modified thermal diffusivity
$D_{m}$	Brownian diffusion coefficient	${\left(\rho C_{p}\right)}_{hnf^{\prime }}$ ${\left(\rho C_{p}\right)}_{f}$	Heat capacity
$E_{c}$	Eckert number	$k_{f},\;k_{hnf}$	Thermal conductivity
$C_{f}$	Surface drag force	$\phi_{1}$	The solid volume fraction of particles
$Nu_{x}$	Nusselt number	ζ	Scaled boundary-layer coordinate
$P_{e}$	Bioconvection Péclet number	$\sigma_{hnf},\;\sigma_{f}$	Electric conductivity
b	Chemotaxis constants	$\lambda_{1}$	Thermal relaxation time
s	Stefan blowing parameter	$\lambda_{1}$	Concentration relaxation time
$L_{e}$	Lewis number	$\gamma_{e}$	Thermal relaxation parameter
$W_{c}$	Maximum cell swimming speed	$\gamma_{c}$	Concentration relaxation characteristic
$L_{b}$	Bio-convection Lewis number	θ	Dimensionless temperature
$h_{h},\;h_{g},\;h_{n}$	Heat, mass, and microorganism transport coefficients, respectively		
Subscripts			
w	The boundary surface	∞	The ambient surface
hnf	Hybrid nanofluid	nf	Nanofluid
